# CT Perfusion Derived rCBV < 42% Lesion Volume Is Independently Associated with Followup FLAIR Infarct Volume in Anterior Circulation Large Vessel Occlusion

**DOI:** 10.3390/diagnostics14080845

**Published:** 2024-04-19

**Authors:** Dhairya A. Lakhani, Aneri B. Balar, Hamza Salim, Manisha Koneru, Sijin Wen, Burak Ozkara, Hanzhang Lu, Richard Wang, Meisam Hoseinyazdi, Risheng Xu, Mehreen Nabi, Ishan Mazumdar, Andrew Cho, Kevin Chen, Sadra Sepehri, Nathan Hyson, Victor Urrutia, Licia Luna, Argye E. Hillis, Jeremy J. Heit, Greg W. Albers, Ansaar T. Rai, Adam A. Dmytriw, Tobias D. Faizy, Max Wintermark, Kambiz Nael, Vivek S. Yedavalli

**Affiliations:** 1 Department of Radiology and Radiological Sciences, Johns Hopkins University, 600 N. Wolfe St., Phipps B100, Baltimore, MD 21287, USA; aneribbalar@gmail.com (A.B.B.); hamza.sleeem@gmail.com (H.S.); hlu3@jhmi.edu (H.L.); rwang93@jhmi.edu (R.W.); mhosein3@jhmi.edu (M.H.); rxu4@jhmi.edu (R.X.); mnabi1@jhmi.edu (M.N.); imazumd1@jhmi.edu (I.M.); acho27@jhmi.edu (A.C.); kchen72@jhmi.edu (K.C.); ssepehr3@jh.edu (S.S.); nhyson1@jh.edu (N.H.); vurruti1@jhmi.edu (V.U.); lluna6@jhmi.edu (L.L.); vyedava1@jhmi.edu (V.S.Y.); 2 Cooper Medical School, Rowan University, Camden, NJ 08103, USA; 3 Department of Biostatistics, West Virginia University, Morgantown, WV 26506, USA; siwen@hsc.wvu.edu; 4 Department of Neurology, Johns Hopkins University, Baltimore, MD 21218, USAargye@jhmi.edu (A.E.H.); 5 Department of Neurology, Stanford University, Stanford, CA 94305, USA; jheit@stanford.edu (J.J.H.); albers@stanford.edu (G.W.A.); 6 Department of Neuroradiology, West Virginia University, Morgantown, WV 26506, USA; ansaar.rai@hsc.wvu.edu; 7 Department of Radiology, Harvard Medical School, Boston, MA 02115, USA; adam.dmytriw@gmail.com; 8 Department of Radiology, Neuroendovascular Division, University Medical Center Münster, 48149 Münster, Germany; tobiasfaizy@web.de; 9 Department of Neuroradiology, MD Anderson Medical Center, Houston, TX 77030, USA; max.wintermark@gmail.com; 10 Division of Neuroradiology, Department of Radiology, University of California San Francisco (UCSF), San Francisco, CA 94143, USA; kambiznael@gmail.com

**Keywords:** relative cerebral blood volume, rCBV < 42%, infarct volume

## Abstract

Pretreatment CT Perfusion (CTP) parameter rCBV < 42% lesion volume has recently been shown to predict 90-day mRS. In this study, we aim to assess the relationship between rCBV < 42% and a radiographic follow-up infarct volume delineated on FLAIR images. In this retrospective evaluation of our prospectively collected database, we included acute stroke patients triaged by multimodal CT imaging, including CT angiography and perfusion imaging, with confirmed anterior circulation large vessel occlusion between 9 January 2017 and 10 January 2023. Follow-up FLAIR imaging was used to determine the final infarct volume. Student t, Mann-Whitney-U, and Chi-Square tests were used to assess differences. Spearman’s rank correlation and linear regression analysis were used to assess associations between rCBV < 42% and follow-up infarct volume on FLAIR. In total, 158 patients (median age: 68 years, 52.5% female) met our inclusion criteria. rCBV < 42% (ρ = 0.56, *p* < 0.001) significantly correlated with follow-up-FLAIR infarct volume. On multivariable linear regression analysis, rCBV < 42% lesion volume (beta = 0.60, *p* < 0.001), ASPECTS (beta = −0.214, *p* < 0.01), mTICI (beta = −0.277, *p* < 0.001), and diabetes (beta = 0.16, *p* < 0.05) were independently associated with follow-up infarct volume. The rCBV < 42% lesion volume is independently associated with FLAIR follow-up infarct volume.

## 1. Introduction

In the setting of an acute ischemic stroke caused by a large vessel occlusion, the cerebral blood volume (CBV) is an essential component of assessing the ischemic core and infarct growth rate. The cerebral blood volume is also a valuable biomarker of the compensatory cerebral response to large vessel occlusion through the collateral routes [1]. This parameter can be measured on pretreatment imaging, but definitions may vary considerably depending on the post-processing used.

In several automated software platforms, the cerebral blood volume is quantified relative to the non-affected hemisphere [1,2,3,4,5,6,7,8]. RapidAI (IschemaView, Menlo Park, CA, USA) is one such software platform with the capability of quantifying cerebral blood volume where the relative cerebral blood volume (rCBV) is derived from the cerebral blood volume values in the region of time to maximum (Tmax) > 6 second lesion area within the affected hemisphere. This parameter is obtained by dividing the average of all cerebral blood volume values from the regions with Tmax > 6 seconds within the ischemic hemisphere by the average of all cerebral blood volume values extracted from all the tissues with normal perfusion, defined as the regions with Tmax ≤ 4 second [1].

A recent study using efficacy and safety of nerinetide for the treatment of acute ischaemic stroke (ESCAPE NA1) trial data determined that increased rCBV < 42% lesion volume was independently associated with worse functional independence at 90 days. The authors concluded that the combined clinical and imaging biomarker model with rCBV < 42% performed better than rCBV < 34% and rCBV < 38% in predicting functional outcomes [9]. However, this study did not directly assess rCBV with respect to follow-up infarct volumes.

Hence, in this study, we aim to assess the relationship of rCBV < 42% lesion volume with follow-up infarct volume on Fluid Attenuated Inversion Recovery (FLAIR) images in anterior circulation acute ischemic stroke secondary to large vessel occlusion patients. We hypothesize that increased rCBV < 42% lesion volumes are associated with larger follow-up infarct volumes.

## 2. Materials and Methods

### 2.1. Study Design

We performed a retrospective analysis of prospectively maintained stroke databases, and we identified consecutive patients from two comprehensive stroke centers from 29 July 2019 to 1 October 2023 who met our inclusion criteria. This study was approved through the Johns Hopkins institutional review board (IRB #00269637) and follows the STROBE checklist guidelines as an observational study.

### 2.2. Study Participants

The inclusion criteria for this study were: (a) diagnostically adequate multimodal pretreatment Computed Tomography (CT) imaging, including non-contrast head CT, CT angiography, and CT perfusion; (b) acute ischemic stroke secondary to large vessel occlusion confirmed on CT angiogram images of the supraclinoid segment of the internal carotid artery, M1-segment of the middle cerebral artery, and proximal M2-segment of the middle cerebral artery [10,11,12]; (c) follow-up diagnostic quality FLAIR images available during the same admission ranging from one to seven days after the symptom onset.

The study was conducted in accordance with the Declaration of Helsinki and the Health Insurance Portability and Accountability Act (HIPAA). Informed consent was waived by the institutional review boards, given the retrospective study design.

The decisions to administer intravenous thrombolysis and/or to perform mechanical thrombectomy were made on an individual basis based on consensus stroke team evaluation per institutional protocols.

### 2.3. Data Collection

Baseline and clinical data were collected through electronic records and stroke center databases for each patient are prospectively collected and maintained, that includes baseline demographics data including age, sex, race, date of admission; clinical markers including hyperlipidemia, hypertension, diabetes, smoking, alcohol, and other; imaging markers on non contrast CT head, CT angiogram and CT perfusion including The Alberta stroke program early CT score (ASPECTS), site of occlusion and different RAPID derived CT perfusion variables; clinical variables including but not limited to premorbid modified Rankin Score, and national institute of health stroke scale; various time parameters including last known well to door time, door to needle time, and door to groin puncture times; and outcome measures on imaging including follow-up infarct volume on T2 fluid-attenuated inversion recovery (T2 FLAIR) imaging.

### 2.4. CTP Image Acquisition

Whole brain pretreatment CT perfusion was performed on the Siemens Somatom Force (Erlangen, Germany) with the following parameters: 70 kVP, 200 effective mAs, rotation time 0.25 s, average acquisition time 60 s, collimation 48 × 1.2 mm, pitch value 0.7, 4D range 114 mm × 1.5 s.

### 2.5. Image Analysis

All the CT perfusion images were assessed by board certified neuroradiologists with 9 years of working experience for the diagnostic adequacy of the CT perfusion, and only those deemed diagnostic adequate were included in the study.

CT perfusion source images were then post processed using commercial RapidAI perfusion software, version 5.2.2 (iSchemaView, Menlo Park, CA, USA), to generate rCBV < 42% lesion volume.

The infarct volume was manually segmented by one board-certified radiologist (5 years of working experience, blinded by the admission CTP imaging and rCBV volumetric maps) on follow-up axial 2D T2 FLAIR brain MRI post-treatment obtained during the same hospital encounter.

### 2.6. Statistical Analysis

The objective of this study is to assess the relationship between rCBV < 42% lesion volume and follow-up FLAIR volume. Categorical data was described using contingency tables, including counts and percentages; continuous variables were summarized with a mean (±Standard Deviation). A student *t* test was used in the data analysis for continuous variables; a Mann Whitney U test was used in the data analysis for ordinal data; and a Chi Square test was used for categorical data.

Spearman’s rank correlation analysis was used to assess the correlation between rCBV < 42% lesion volume and follow-up T2 FLAIR volume.

Univariable and multivariable linear regression models were used to estimate the relationship between rCBV < 42% lesion volume and follow-up FLAIR volume.

The multivariable linear regression model took into account confounding variables: age, sex, race, hypertension, hyperlipidemia, diabetes mellitus, heart disease, atrial fibrillation, occlusion segment, prior history of transient ischemic attack or stroke, intravenous tissue-type plasminogen activator (IV tPA), admission national institute of health stroke scale, premorbid modified Rankin score (mRS), Alberta Stroke Program Early CT Score (ASPECTS), and the modified treatment in cerebral infarction (mTICI) score. The outcomes were reported as a regression slope or regression coefficient with a 95% confidence interval and a *p*-value. Statistically significant analysis was described as *p* ≤ 0.05, *p* < 0.01, and *p* < 0.001.

## 3. Results

A total of 158 consecutive patients (median age: 68 years, 52.5% female) met our inclusion criteria. The majority of our cohort was either Caucasian (*n* = 79, 50%) or African American (*n* = 70, 44.3%). In our cohort, 126 (79.8%) had hypertension, 79 (50.0%) had diabetes, and 74 (46.8%) had a smoking history (Table 1).

Of the 158 patients, 114 (72.2%) had M1 segment occlusion, 32 (20.3%) had proximal M2 segment occlusion, and 12 (7.6%) had distal ICA supraclinoid segment occlusion (Table 2).

The median relative cerebral blood flow (rCBF) < 30% lesion volume was 7.5 mL (0–38 mL). The median duration between symptom onset and follow-up T2 FLAIR imaging was 2 days (Interquartile range: 1–3 days), and the median door to CT time was 30 min (21–46 min) (Table 3).

In total, 51 patients (32.3%) received intravenous tissue plasminogen activator (IV tPA), and 130 patients (82.3%) underwent mechanical thrombectomy (MT) (Table 4).

Patient demographics are described in Table 1, imaging parameters are described in Table 2, stroke treatment details are presented in Table 3, and management-related time parameters are described in Table 4.

The median follow-up T2 FLAIR infarct volume was 32.25 mL (Interquartile range: 7.12–114.52 mL), and the median rCBV < 42% lesion volume was 6.0 mL (Interquartile range: 0.0–33.0 mL) (Table 2).

**Figure 1:**
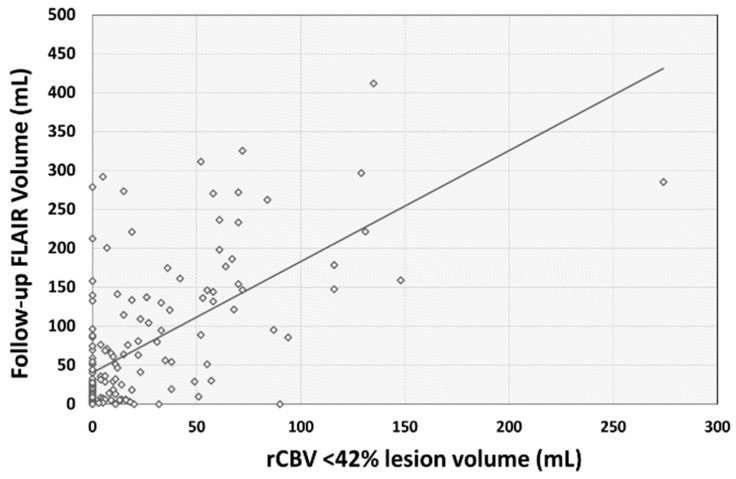
Scatter plot showing distribution of rCBV < 42% lesion volume with follow-up FLAIR infarct volume. A significant positive correlation was observed between rCBV < 42% lesion volume and follow-up FLAIR infarct volume (ρ = 0.56, *p* < 0.001, 95% CI: 0.44–0.66).

A significant positive correlation was observed between rCBV < 42% lesion volume and follow-up FLAIR infarct volume (ρ = 0.56, *p* < 0.001, 95% CI: 0.44–0.66) (Figure 1).

On linear regression analysis, rCBV < 42% lesion volume was associated with follow-up FLAIR infarct volume (unadjusted beta: 1.48, *p* < 0.001). Multivariable linear regression analysis taking into account various confounding markers showed that rCBV < 42% lesion volume (adjusted beta: 0.60, *p* < 0.001), ASPECTS (adjusted beta: −0.21, *p* < 0.01), mTICI (adjusted beta: −0.28, *p* < 0.001), and diabetes (adjusted beta: 0.16, *p* < 0.05) were independently associated with follow-up FLAIR infarct volume (Table 5).

## 4. Discussion

In this study of acute ischemic stroke secondary to anterior circulation large vessel occlusion in patients undergoing multimodal admission CT imaging triage, we found that rCBV < 42% lesion volume was independently associated with follow-up infarct volume. To our knowledge, thresholds of rCBV for correlating with follow-up infarct volume still remain sparse. The study validates rCBV < 42% specifically as an additional prognostic biomarker of follow-up infarct volume in acute ischemic stroke secondary to large vessel occlusion.

The rCBV thresholds quantify cerebral blood volume in the region of ischemia (with Tmax > 6 s) relative to the cerebral blood volume values in unaffected regions (with Tmax ≤ 4 s), thereby providing an estimate of hypoperfused brain tissue [1]. The normal homeostatic response to ischemia is vasodilation, where increased oxygen extraction is facilitated by increasing cerebral blood volume through the collateral routes [1]. Conversely, the absence of sufficient compensatory vasodilation manifests as decreased cerebral blood volume, leading to a higher infarct growth rate as compared to those patients with a robust compensatory response [13].

A handful of prior studies have assessed rCBV as a biomarker of infarct progression. Mokin et al. [14] using Solitaire with the Intention for Thrombectomy as Primary Endovascular Treatment (SWIFT PRIME) trial data of small core (≤50 mL) found that rCBV thresholds can reliably predict infarct volume on follow-up CT or FLAIR at 27-h in patients who achieved complete reperfusion. However, patients who did not achieve complete reperfusion were excluded. Furthermore, Arenillas et al. [1], also using SWIFT PRIME trial data, reported that lower rCBV predicted higher infarct growth following mechanical thrombectomy (*p* = 0.049) and in those who had successful reperfusion (*p* = 0.0038). While the results of rCBV in predicting infarct growth using SWIFT PRIME trial data validate rCBV as a reliable biomarker, patients with large cores (>50 mL) were nevertheless excluded. Our study differs from Mokin et al. and Arenillas et al. in that we also include patients who did not successfully reperfuse, did not undergo mechanical thrombectomy, and had large infarct cores (≥50 mL), as these groups may have been underrepresented in these prior investigations [1].

Despite these differing inclusion criteria, our results nevertheless corroborate these prior studies in demonstrating that higher volumes of rCBV < 42% result in larger follow-up infarct volumes. Our findings again support the physiological mechanism of lower rCBV capturing the degree of compensation through collateral routes, where patients who are unable to generate a sufficient compensatory response have higher infarct volumes.

Our results also demonstrate independent associations with other well-established parameters. Both the Alberta stroke program early CT score (ASPECTS) and modified treatment in cerebral infarction (mTICI) score were independently associated with follow-up infarct volume, demonstrating the strong relationships between initial ischemic core and reperfusion status and follow-up infarct volume [15,16,17,18,19].

Limitations of our study include the retrospective study design and that follow-up T2 FLAIR volume may be overestimated due to edema during days 2–5 or underestimated due to the continued evolution of ischemic lesions [20]. However, our study is strengthened by the sample size of 158 patients with anterior circulation large vessel occlusion that were derived from two comprehensive stroke centers serving different demographics.

## 5. Conclusions

In conclusion, our results validate the potential role of rCBV < 42% lesion volume in estimating follow-up infarct volume. More recently, large core trials such as the TENSION (The Efficacy and Safety of Thrombectomy in Stroke with extended lesion and extended time window) trial [21] showed the benefit of mechanical thrombectomy in large vessel occlusion cases, irrespective of ischemic core volume, on pretreatment imaging. Hence, CT perfusion-based rCBV < 42% lesion volume may be used as an adjunct marker in mechanical thrombectomy triage of especially complex acute ischemic stroke cases secondary to large vessel occlusion, where the decision is more nuanced. Future studies are needed to expand our understanding of the adjunct role of rCBV < 42% with other similar pretreatment imaging-based markers in clinical evaluation and decision-making in patients with acute ischemic stroke caused by a large vessel occlusion, particularly in the large core subset of patients, those who did not underwent mechanical thrombectomy, and those with unsuccessful recanalization.

## Figures and Tables

**Table 1:** Demographics of the study population.

Study Demographics (*n* = 158)	Median (Interquatile Range) or Number (Percentage)
Age	68 (60–77)
Sex	
Female	83 (52.53%)
Male	75 (47.47%)
Race	
African American	70 (44.30%)
Caucasian	79 (50.00%)
Asian	4 (2.53%)
Others	5 (3.16%)
Comorbidities	
Hypertension	126 (79.75%)
Hyperlipidemia	79 (50.00%)
Diabetes Mellitus	41 (25.95%)
Heart Disease	77 (48.73%)
Atrial Fibrillation	57 (36.08%)
Smoking	74 (46.84%)
Prior stroke or transient Ischemic Attack	30 (18.99%)

**Table 2:** Imaging findings in the study cohort.

Study Cohort (*n* = 158)	Median (Interquatile Range) or Number (Percentage)
Vessel Occluded	
M1	114 (72.15%)
Proximal M2	32 (20.25%)
Supraclinoid ICA	12 (7.59%)
Alberta Stroke Program Early CT Score (ASPECTS)	
0	2 (1.27%)
1	1 (0.63%)
2	4 (2.53%)
3	3 (1.90%)
5	10 (6.33%)
6	8 (5.06%)
7	12 (7.59%)
8	27 (17.09%)
9	22 (13.92%)
10	69 (43.67%)
CT Perfusion Parameter	
rCBV < 42% (mL)	6.00 (0.00–33.00)
rCBF < 30% (mL)	7.50 (0.00–38.00)
Tmax > 6 s (mL)	110.00 (66.00–161.00)
MRI Parameter	
FLAIR Volume (mL)	32.25 (7.12–114.52)

**Table 3:** Time parameters related to stroke management.

Study Cohort (*n* = 158)	Median (Interquatile Range) or Number (Percentage)
Symptom onset to door time in minutes	66.00 (44.00–105.00)
Door-to-CT time in minutes	30.00 (21.00–46.00)
Door-to-needle time in minutes	59.00 (47.50–82.50)
Door-to-groin puncture time in minutes	178.00 (133.00–221.00)
Door-to-recanalization time in minutes	32.00 (24.00–53.00)
Symptom onset to MRI time in days	2.00 (1.00–3.00)

**Table 4:** Treatment details of the study cohort.

Study Cohort (*n* = 158)	Median (Interquatile Range) or Number (Percentage)
Intravenous tissue-type plasminogen activator (IV tPA)	51 (32.28%)
Mechanical thrombectomy (MT)	130 (82.28%)
Admission NIH Stroke Scale	15 (10–20)
Premorbid Modified Rankin Score (mRS)	
0	100 (65.79%)
1	20 (13.16%)
2	11 (7.24%)
3	19 (12.50%)
4	1 (0.66%)
5	1 (0.66%)
Modified treatment in cerebral infarction (mTICI)	
0	6 (4.96%)
1	1 (0.83%)
2A	4 (3.31%)
2B	27 (22.31%)
2C	17 (14.05%)
3	66 (54.55%)

rCBV < 42% lesion volume and follow-up infarct volume.

**Table 5:** Linear regression analysis with follow-up FLAIR infarct volume as the outcome.

Variables	Unstandardized Coefficients		Multivariable Regression Model			
	Unadjusted Beta	Standard Error	Adjusted Beta	Lower Bound	Upper Bound	*p* Value
rCBV < 42%	1.48	0.18	0.60	1.133	1.83	<0.001
Age	−0.56	0.40	−0.10	−1.36	0.23	0.16
Sex	−4.33	10.56	−0.03	−25.27	16.61	0.68
Race	3.78	7.74	0.03	−11.58	19.13	0.63
Hypertension	18.14	14.21	0.09	−10.06	46.34	0.21
Hyperlipidemia	−11.68	10.75	−0.07	−33.01	9.65	0.28
Diabetes Mellitus	28.39	12.27	0.16	4.04	52.73	<0.05
Heart Disease	0.67	12.08	0.004	−23.29	24.63	0.96
Atrial Fibrillation	5.17	12.24	0.03	−19.12	29.46	0.67
Occlusion Segment	−0.81	3.56	−0.02	−7.87	6.26	0.82
Prior transient Ischemic Attack or stroke	7.58	14.62	0.04	−21.43	36.58	0.61
Intravenous tissue-type plasminogen activator (IV tPA)	−22.00	11.69	−0.13	−45.15	1.14	0.06
Admission NIH Stroke Scale	0.89	0.83	0.08	−0.76	2.55	0.29
Premorbid Modified Rankin Score (mRS)	−0.48	5.29	−0.007	−10.98	10.02	0.93
Alberta Stroke Program Early CT Score (ASPECTS)	−9.37	2.99	−0.21	−15.29	−3.44	<0.01
The modified treatment in cerebral infarction (mTICI) score	−14.58	3.40	−0.28	−21.33	−7.825	<0.001

## Data Availability

The datasets used and/or analyzed during the current study are available from the corresponding author on reasonable request.
